# Properties of the Sickness Questionnaire in an Australian sample with chronic medically unexplained symptoms

**DOI:** 10.1016/j.bbih.2020.100059

**Published:** 2020-03-10

**Authors:** Anna Andreasson, David McNaughton, Alissa Beath, Karin Lodin, Rikard K. Wicksell, Mats Lekander, Michael P. Jones

**Affiliations:** a Department of Psychology, Macquarie University, Australia; b Stress Research Institute, Stockholm University, Sweden; c Department of Clinical Neuroscience, Karolinska Institutet, Sweden; d Department of Neurobiology, Care Sciences and Society, Karolinska Institutet, Sweden

**Keywords:** Sickness behavior, Sickness questionnaire, Medically unexplained symptoms

## Abstract

Sickness behavior including malaise, fatigue and increased pain sensitivity is thought to be adaptive and facilitate recovery from disease. However, it may also reduce functioning and health if symptoms persists, which is why validated instruments for its assessment are needed. We evaluated the English translation of the Sickness Questionnaire (SicknessQ) in an Australian population of 156 participants with high level of persistent musculoskeletal pain and/or gastrointestinal symptoms without an organic explanation. The SicknessQ total score had an adequate model fit and no other models were found to fit data better. The SicknessQ correlated most strongly with fatigue, stress, anxiety and depression, which explained 62% of the variance in SicknessQ, but not with physical functioning. The mean score (8.9; 95 %CI: 8.0–9.8) was in between those previously reported in a general population sample and in primary care patients. In conclusion, the evaluation of the English version of the SicknessQ in an Australian sample with significant, chronic unexplained medical symptoms supports the use of the English version of the total SicknessQ score as an overall measure of sickness behavior.

## 1 Introduction

Sickness behavior is a set of symptoms occurring in response to an immune challenge and include malaise, fatigue, anxiety, anhedonia and increased pain sensitivity (Dantzer, 2008). Sickness behavior is thought to be adaptive and facilitate recovery from disease but may also contribute to ill health if symptoms persist. This long term perspective of immune activated sickness behavior has been increasingly explored, including in depression (Dantzer, 2008, 2018), pain (Karshikoff, 2016) and in relation to general health appraisal (Andreasson, 2019; Lodin, 2019). In spite of the clear clinical relevance, there is no validated self-report instrument for sickness behavior. Recently, the Sickness Questionnaire (SicknessQ) was developed for this purpose (Andreasson, 2018). Items from the original item pool that reflected experimentally induced sickness behavior by injection of lipopolysaccharide (LPS) were included in the psychometric evaluation based on a primary care sample, which resulted in 10 items being retained which all pertained to a single dimension of sickness behavior (Andreasson, 2018).

The main use to which the SicknessQ instrument has been applied thus far has been to quantify symptoms of sickness behavior in experimental studies with transient immune activation using LPS (Lekander, 2016; Henderson, 2017; Lasselin, 2017, Lasselin et al., 2018a, 2018b, 2020; Marraffa, 2017; Regenbogen, 2017; Axelsson, 2018; Lasselin, 2018; Andreasson, 2019). SicknessQ has also been used to quantify and compare the degree of sickness behavior symptoms between different patient populations and comparison groups (Jonsjö, 2020), to evaluate treatment response to cognitive behavioral therapy in patient with health anxiety (Hedman-Lagerlof, 2017) and chronic stress (Lindsater, 2018), and in an observational study on self-rated health and health anxiety (Lodin, 2019). In addition, the English translation of the SicknessQ has been used to investigate how the experience of sickness behavior is influenced by demographics and sociocultural norms and values (Shattuck, 2020). However, the English version of the SicknessQ and its application in clinical populations with expected elevated levels of sickness needs further validation.

The aim of the present study was to assess sickness behavior in an Australian population with significant, chronic medically unexplained symptoms, i.e. high levels of persistent symptoms of pain, fatigue and distress without any demonstrable disease or illness using the English version of the SicknessQ in order to evaluate the questionnaire in a population with more chronic symptoms than in the original validation sample (Andreasson, 2018).

## 2 Methods

### 2.1 Participants

Participants with a high level of persistent musculoskeletal pain and/or gastrointestinal symptoms were recruited through chiropractic clinics (for chronic musculoskeletal pain), gastroenterology clinics (for chronic functional gastrointestinal disorders/FGIDs), and from undergraduate university students who had previously reported high levels of musculoskeletal pain and/or gastrointestinal symptoms. Participants were excluded if they reported any organic disease which may explain symptom reporting e.g. inflammatory bowel disease or inflammatory arthritis. A total of 158 participants (84% women) primarily within an 18–25 age range (76%) agreed to participate in the study and completed the online questionnaire. Criteria for classification of FGIDs was made using the ROME III modular questionnaire for irritable bowel syndrome and functional dyspepsia (Drossman, 2006) and chronic musculoskeletal pain was determined in those who indicated low back pain, neck pain or headache, which occurred most days for at least 3 months (Merskey, 1994). Twenty-three (15%) participants were classified with FGIDs only, 43 (28%) participants with a chronic musculoskeletal pain and 52 (33%) with a co-occurring FGID and chronic musculoskeletal pain. The remaining 40 (26%) experienced varying degrees of musculoskeletal pain and gastrointestinal symptoms without fulfilling the criteria for FGID or chronic musculoskeletal pain.

### 2.2 SicknessQ

The Sickness Questionnaire includes 10 statements of sickness symptoms (“I want to keep still”, “My body feels sore”, “I wish to be alone”, “I don’t wish to do anything at all”, “I feel depressed”, “I feel drained”, “I feel nauseous”, “I feel shaky”, “I feel tired”, “I have a headache”) rated on a 4 point scale from disagree (0) to agree (3) (Andreasson, 2018), see Appendix. The questionnaire was developed in Swedish and translated to English by two independent bilingual native English speakers living in Sweden (one scientific editor and one primary school principal) and the resulting translation is the consensus of the two independent translations. The questionnaire was further back translated to Swedish by two independent bilingual native Swedish speakers who had lived in English speaking countries (scientists). The Swedish back translation did not differ from the Swedish original ensuring that the meaning had not been altered during the translation process.

### 2.3 Concurrent criterion validity measures

**Self-rated health** was assessed using the question ”How do you rate your general state of health?” rated on a 5-point scale from very good (1) to very poor (5). **Mental and physical functioning** were assessed using the mental and physical health subscales of Short form 12 (SF 12) (Brazier, 1992). **Anxiety, Depression and Stress** were assessed using the subscales for anxiety, depression and stress from the Depression, Anxiety and Stress – 21 questionnaire (DASS-21) (Lovibond, 1995). **Fatigue.** The Chalder Fatigue Scale contains aspects of both physical (e.g., “Do you lack energy?”) and mental fatigue (“Do you have difficulties concentrating?”) (Cella, 2010). **Somatization.** The Patient Health Questionnaire (PHQ)-15 assesses the level of somatization for symptoms such as stomach ache, dizziness and chest pain (Kroenke, 2002). **Neuroticism.** The International Personality Item Pool neuroticism scale assess the tendency to experience distressing or negative emotions (Goldberg, 2006). **Pain catastrophizing.** The Pain Catastrophizing Scale (PCS) assesses catastrophic thinking related to pain. It includes items such as “I worry all the time about whether the pain will end” (Sullivan, 1995). **Kinesiophobia.** The Tampa Scale of Kinesiophobia (TSK) is designed to measure fear avoidance behavior as it relates to movement (Roelofs, 2004). **Gastrointestinal symptoms.** The Gastrointestinal Symptom Rating Scale (GSRS) assesses common symptoms of the gastrointestinal tract (Svedlund, 1988).

### 2.4 Statistics

Firstly, a confirmatory factor analysis (CFA) of the 10 item single dimension structure of the SicknessQ, as identified previously (Andreasson, 2018), was performed. While not reported in our original population, we also report the equivalent fit statistics for this model for the original Swedish sample here. Secondly, because the model fit did not fulfill all criteria for perfect fit (see section 3) and we did not have an a priori hypothesis about how the model should be recast to fit the data better, we performed a principle components analysis (PCA) of the 10 items for guidance to propose an alternate model. Thirdly, the suggested item structures from the PCA were then fitted to the data using CFAs separately to quantify and compare the degree to which they were supported by the data (Table 1). Any resulting model was required to both reproduce the observed correlation matrix of observed variables (fit well) and also be theoretically sound (for example, consistent with brain regions being involved in different types of sickness behaviors and somatic symptoms developing earlier than depression during immune activation (Dantzer, 2008)). The concurrent criterion validity of the chosen solution was further evaluated by correlating total and revised factor scores with concurrent criterion validity measures (Table 2). All analyses were performed in Stata 15.1.

**Table 1:** Standardized coefficients and fit statistics for the three factor solutions from the principal component analysis.

Item (questionnaire item number)	Single factor	95% CI	Two factor	95% CI	Three factor	95% CI
	Standardized Coefficient		Standardized Coefficient		Standardized Coefficient	
I want to keep still (1)	0.34	0.18-0.49	0.43^b^	0.29-0.56	0.52^c^	0.36-0.68
My body feels sore (2)	0.31	0.16-0.47	0.39^a^	0.24-0.54	0.32^b^	0.27-0.57
I wish to be alone (3)	0.56	0.46-0.69	0.71^b^	0.61-0.80	0.66^a^	0.54-0.76
I don’t wish to do anything at all (4)	0.58	0.46-0.71	0.82^b^	0.71-0.93	0.78^c^	0.62-0.95
I feel depressed (5)	0.72	0.63-0.82	0.88^b^	0.78-0.97	0.79^a^	0.70-0.87
I feel drained (6)	0.76	0.67-0.84	0.80^a^	0.71-0.90	0.83^b^	0.73-0.93
I fell nauseous (7)	0.71	0.61-0.81	0.64^a^	0.52-0.76	0.78^a^	0.58-0.79
I feel shaky (8)	0.70	0.60-0.79	0.65^a^	0.53-0.77	0.70^a^	0.60-0.80
I feel tired (9)	0.58	0.46-0.71	0.66^a^	0.49-0.73	0.74^b^	0.61-0.86
I have a headache (10)	0.54	0.42-0.69	0.61^a^	0.49-0.73	0.61^b^	0.49-0.73
**Measure of fit (reference ideal fit)**						
Chi^b^/df (<5.0)	1.66		2.24		1.78	
RMSEA (>0.05)	0.065		0.089		0.071	
Residual Chi-Square test p-value (p ​> ​0.05)	0.015		0.001		0.007	
The comparative fit index (>0.95)	0.968		0.934		0.963	
Tucker-Lewis index (>0.95)	0.950		0.905		0.940	
Akaike information criterion (AIC)	3598.651		3616.066		3602.548	

CI: confidence interval.

RMSEA: Root mean square error of approximation.

a First factor.

b Second factor.

c Third factor.

**Table 2:** Correlations coefficients (Spearman’s Rho) between SicknessQ and concurrent criterion validity measures.

	Mean (SD)	Min-max	Correlation with SicknessQ
SicknessQ	8.91 (5.93)	0–30	–
Self-rated health	2.69 (0.76)	1–5	0.34∗∗
Physical functioning (SF-12)	43.89 (6.04)	0–100	0.02
Mental functioning (SF-12)	47.96 (8.69)	0–100	−0.37∗∗
Anxiety (DASS-21 subscale)	12.18 (4.06)	7–28	0.55∗∗
Depression (DASS-21 subscale)	12.59 (4.27)	7–28	0.55∗∗
Stress (DASS subscale)	14.68 (4.06)	7–28	0.56∗∗
Fatigue (Chalder’s)	19.46 (5.47)	0–33	0.60∗∗
Somatization (PHQ-15)	17.39 (3.32)	0–30	0.54∗∗
Neuroticism (IPIP)	9.97 (0.18)	0–20	0.54∗∗
Pain catastrophizing (PCS)	26.44 (11.40)	0–52	0.32∗∗
Kinesophobia (TSK)	33.99 (6.26)	17–68	0.38∗∗
GI symptoms (GSRS)	35.01 (13.04)	15–105	0.35∗∗
Explained variance			0.62

SF-12: Short form scale 12.

DASS-21: Depression, Anxiety and Stress – 21 questionnaire (DASS-21).

PJQ: The Patient Health Questionnaire.

IPIP: The International Personality Item Pool neuroticism scale.

PCS: The Pain Catastrophizing Scale.

TSK: The Tampa Scale of Kinesiophobia.

GSRS: The Gastrointestinal Symptom Rating Scale.

∗p ​< ​0.01, ∗∗p ​< ​0.001.

#### 2.4.1 Confirmatory factor analysis

Covariance terms which would improve model fit substantively (reduction in residual Chi-Square statistic >10) were included in the CFA and were identified using modification indices. The fit of the models was evaluated using the residual Chi-Square test (ideally p ​> ​0.05), the ratio Chi-Square/degrees of freedom (df) (ideally <5.0), the comparative fit index (CFI, ideally >0.95) and the Tucker-Lewis index (TLI, ideally >0.95) and Root mean square error of approximation (RMSEA, ideally <0.05) (Schermelleh-Engel, 2014). Akaike information criterion (AIC) was used to quantitatively compare fit between models. Missing data were excluded listwise leaving 156 participants in the analyses.

#### 2.4.2 Principal component analysis

The PCA included all 10 items, was based on a Pearson correlation matrix and utilized an oblimin rotation and an eigenvalue cut off of 1 to identify the latent factor(s) to be consistent with the original Swedish validation procedure (Andreasson, 2018). Missing data were excluded listwise.

#### 2.4.3 Correlation with concurrent criterion validity measures

Correlations between total and factor scores with concurrent criterion validity measures were calculated using Spearman’s correlation due to the non-Normal distribution of some measures. The total variance in the SicknessQ explained by the combination of all criteria constructs was calculated through multiple regression. Missing data were excluded pairwise.

## 3 Results

The single factor solution showed a good fit meeting most fit criteria with a residual Chi-Square test p ​= ​0.015, Chi-Square/df ​= ​1.66, CFI ​= ​0.97, TLI ​= ​0.95, RMSEA ​= ​0.065 and AIC ​= ​3598.651 (Table 1). In the original Swedish sample, the single factor solution had a Chi-Square test p-value ​= ​0.003, Chi-Square/df ​= ​1.79, CFI ​= ​0.95, TLI ​= ​0.94 and RMSEA ​= ​0.069. As the fit was imperfect on some measures, we decided to explore if there were better solutions than the single factor solution in the Australian population and a PCA was conducted. The initial PCA suggested a 3-component solution. However, the third component included only two items, had an Eigenvalue of 1.006 and had limited theoretical justification. Given the tendency for PCA to produce more components than actually exist we investigated both the 3-factor solution and a solution restricted to 2 factors for parsimony using CFA. There was no improvement in AIC for the 2-factor or 3-factor solution compared to the single factor solution (Table 1) from which we conclude that a multidimensional structure offers no clear advantage over a single dimensional structure. Hence, the single factor solution was used in further evaluations of concurrent criterion validity.

The SicknessQ correlated statistically significantly with all concurrent criterion validity measures (Spearman’s rho range 0.32–0.63, Table 2) and most strongly with fatigue, stress, anxiety and depression. The exception was physical functioning scale from SF-12 which was not statistically significantly correlated with SicknessQ. The explained variance was 0.62 indicating that slightly more than half of the SicknessQ construct could be accounted for by pre-existing constructs and hence that the SicknessQ contains a significant amount of new information.

**Fig. 1:**
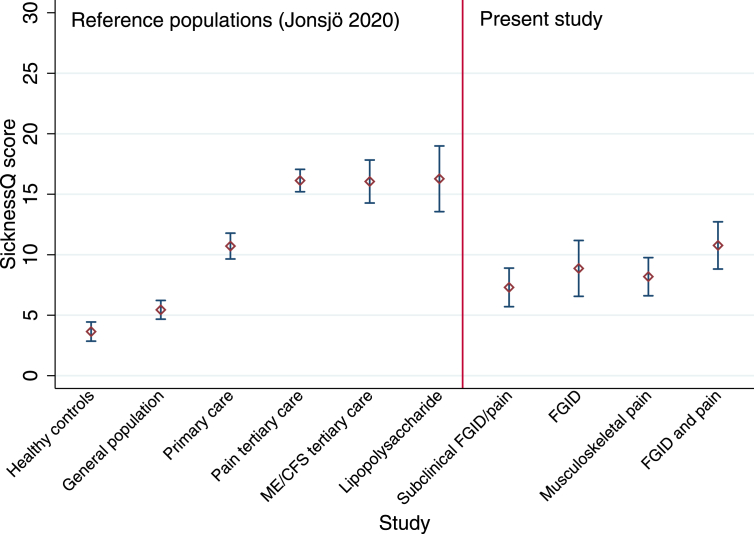
Comparision of mean SicknessQ score from present study and previously published mean SicknessQ scores with 95 % confidence intervals. FGID: functional gastrointestinal disorders. ME/CFS: chronic fatigue syndrome. Lipopolysaccharide: 0.6 ng lipopolysaccharide/kg body weight at peak immune activation 90 ​min post injection.

The average score was 8.91 (95 %CI: 7.98–9.84) in the total sample, the highest scores were reported by participants with both FGID and chronic musculoskeletal pain (mean ​= ​10.8; 95%CI: 8.82–12.7) and the lowest score was found in participants with symptoms not reaching the threshold for FGID or chronic musculoskeletal pain (mean ​= ​7.30; 95%CI: 5.70–8.90) while participants fulfilling the criteria for either FGID (mean ​= ​8.87; 95% CI: 6.56–11.2) or chronic musculoskeletal pain (mean ​= ​8.19; 95%CI: 6.61–9.76) reported average scores in between those two groups. In Fig. 1, the mean scores of the subgroups in the present sample are compared to mean scores of previously published populations for reference (Jonsjö, 2020).

## 4 Discussion

The aim of the present study was to assess sickness behavior in an Australian population with chronic medically unexplained symptoms using the English version of the SicknessQ in order to evaluate the questionnaire in a population with medically unexplained symptoms of a more chronic nature than in the original validation sample of primary care patients to a large degree presenting with an acute infection. The single factor solution showed good fit, equal to the fit in the original validation data, and no other model fit the data better. Except for the physical functioning subscale of SF-12, SicknessQ correlated statistically significantly with all scales (Spearman’s rho range 0.32–0.63) and most strongly with fatigue, stress, anxiety and depression measures. The explained variance was 0.62, indicating that the SicknessQ total scale clearly relates to constructs it would be expected to relate to, indicating good concurrent criterion validity, but that the scale also contributes substantial new information over and above those existing measures.

The average SicknessQ score found in the present study (mean ​= ​8.9) is just below the average in the Swedish primary care population presenting with acute symptoms (mean ​= ​10.7) used for the original validation (Andreasson, 2018), but higher than in a general population (mean ​= ​5.4) (Jonsjö, 2020), see Fig. 1. This level of sickness behavior may be expected as the participants in the present study includes both patients recruited in health care settings and individuals with chronic symptoms recruited from outside of healthcare. The lower level of sickness behavior in the present study compared to the reference populations of patients with pain and chronic fatigue syndrome referred to treatment in tertiary care which report similar levels of sickness behavior compared to healthy individuals with experimentally induced sickness behavior by injection of LPS (Jonsjö, 2020) is also reasonable given the severity of symptoms in the latter groups.

In the present study, no significant association were found between sickness behavior and physical functioning. This is in concordance to the findings in other populations with chronic symptoms (Jonsjö, 2020) where a significantly weaker association between SicknessQ and self-rated health and both physical and mental functioning was found in patients with chronic pain than in the general population, and no association was found between SicknessQ and self-rated health and functioning in patients with chronic fatigue syndrome. We hypothesize that patients with persistent high level of sickness behavior may develop coping strategies over time in order to reduce the influence of sickness behavior on perceived global health and functioning (Jonsjö, 2020).

The present study consisted of individuals with medically unexplained symptoms of a more chronic nature as compared to the sample used for the original psychometric evaluation consisting of patients from a primary care drop-in clinic with a large proportion (42%) presenting with an acute infection. In addition, cultural differences in the interpretation of sickness symptom wording might be expected as there is a recent report suggesting that sickness behavior symptom reporting may be shaped by multiple beliefs and social norms across different demographic groups (Shattuck, 2020). However, the confirmation of the one factor solution as previously found in the original validation supports the hypothesis that the SicknessQ behaves similarly between the two populations despite the difference in chronicity of symptoms, language and potentially other cultural factors.

The SicknessQ was developed based on a model of acute transient immune activation and validated in a primary care sample with a large proportion with an acute infection, and the current study adds important new information in that it appears to also be relevant to populations suffering from chronic and relapsing/remitting disorders. This extension of the instrument’s validity to also disorders of a more chronic nature makes it suitable for comparing levels of sickness behavior between distinct patient groups. That the instrument also differentiates chronic disease groups from relatively healthy individuals further expands its utility as a measure of behavioral changes in response to various types of health problems. Future studies should evaluate if there is a change in SicknessQ in patients with an inflammatory disease such as atopic disease and inflammatory bowel disorder within and without active disease periods where it can also be compared to validated disease specific symptom questionnaires.

In conclusion, the properties of the English version of the SicknessQ in an Australian sample with significant, chronic unexplained medical symptoms supports the use of the English version of the total SicknessQ score as an overall measure of sickness behavior.

## Author contributions

AA: Conceptualization; Data curation; Formal analysis; Writing – original draft, Writing – review and editing.

DM: Investigation; Data curation; Formal analysis; Writing – review and editing.

AB: Data curation; Project administration; Supervision; Writing – review and editing.

KL: Investigation; Data curation; Writing – review and editing.

RW: Writing – review and editing.

ML: Conceptualization; Writing – review and editing.

MPJ: Conceptualization; Methodology; Formal analysis; Supervision; Writing – original draft, Writing – review and editing.

All authors give final approval of the submitted version.

## Funding

This research did not receive any specific grant from funding agencies in the public, commercial, or not-for-profit sectors.

## Ethical approval

All procedures performed in studies involving human participants were in accordance with the ethical standards of Macquarie University Human Research and Northern Sydney Local Health District ethics committees (reference number 5201500188) and with the 1964 Helsinki declaration and its later amendments or comparable ethical standards.

## Informed consent

Informed consent was obtained from all individual participants included in the study.

## Declaration of competing interest

None.

## Figures and Tables

**Table:** 

		Disagree	Agree somewhat	Mostly agree	Agree
1.	I want to keep still	0	1	2	3
2.	My body feels sore	0	1	2	3
3.	I wish to be alone	0	1	2	3
4.	I don’t wish to do anything at all	0	1	2	3
5.	I feel depressed	0	1	2	3
6.	I feel drained	0	1	2	3
7.	I feel nauseous	0	1	2	3
8.	I feel shaky	0	1	2	3
9.	I feel tired	0	1	2	3
10.	I have a headache	0	1	2	3

## Appendix

### Sickness Questionnaire.

INSTRUCTION: Read the statements below and then circle the number that best corresponds to *how you currently feel, in this very moment*. There are no right or wrong answers. Don’t use too much time at each statement, just pick the answer you think best describes how you feel right now.
